# Acute Coronary Syndrome and COVID-19: A Case Report of Refractory Hypercoagulability

**DOI:** 10.7759/cureus.13675

**Published:** 2021-03-03

**Authors:** Fatima Inam, Priyanka R Singh, Farhan Khalid, Aqib Javed, Anuj R Shah

**Affiliations:** 1 Internal Medicine, King Edward Medical University, Lahore, PAK; 2 Internal Medicine, St Luke's Baylor Hospital, Houston, USA; 3 Internal Medicine, King Edward Medical University, Mayo Hospital, Lahore, PAK; 4 Surgery, Ross University School of Medicine, Lynbrook, USA; 5 Internal Medicine, St Mary's General Hospital, Passaic, USA

**Keywords:** myocardial ischemia, covid-19, dvt, thrombosis

## Abstract

Novel coronavirus disease 2019 (COVID-19) is known to cause severe bilateral pneumonia and acute respiratory distress syndrome (ARDS), leading to difficulty breathing requiring mechanical ventilation and ICU management. In many patients, it has been found to cause severe hypercoagulability. We present a case of COVID-19 positive patient who developed myocardial infarction (MI) despite being on multiple anticoagulants. A 51-year-old, Middle-Eastern male diabetic patient presented to the emergency room with complaints of sudden onset left leg pain, paresthesias, and swelling for one day. On physical examination, the left leg was cool to touch from forefoot to mid-calf, with noticeable mottling over the forefoot and a nonpalpable dorsalis pedis. The patient was started on therapeutic enoxaparin and diltiazem in ED. Chest X-ray showed bilateral pulmonary infiltrates beginning peripherally and COVID-19 pneumonitis. The patient underwent a mechanical thrombectomy and was loaded with aspirin/clopidogrel, heparin drip, and enoxaparin. Despite being on triple anticoagulation, the patient had new-onset STEMI and elevated troponin levels. On angiography, the patient was found to have occluded mid-left anterior descending, most likely from acute on chronic thrombosis related to the patient’s COVID-19 status. As flow could not be re-established, the patient was kept on long-term protective anticoagulation-triple therapy (an oral anticoagulant and dual antiplatelet therapy) and received pulmonary care for COVID-19 infection. The patient was discharged on long-term triple anticoagulation and COVID-19 precautions with scheduled retesting and follow-up.

## Introduction

Novel coronavirus disease 2019 (COVID-19), also known as severe acute respiratory syndrome coronavirus 2 (SARS-CoV-2), is an enveloped, nonsegmented positive-sense RNA virus belonging to the beta-coronaviridae family. This virus is known to cause severe bilateral pneumonia and acute respiratory distress syndrome (ARDS), leading to difficulty breathing requiring mechanical ventilation and ICU management. In many patients, it has been found to cause severe hypercoagulability. It was found to develop a prothrombotic state, leading to arterial and venous thrombus formation [1]. Many studies on pathophysiology have been published but the condition is poorly understood. In a study conducted in Wuhan, China, the incidence of venous thromboembolism in the patients admitted in the ICU was 25% [2]. In the Netherlands, a study was done on 180 ICU patients with COVID-19 pneumonia; the incidence of thrombotic complications was found to be 31%, with arterial prothrombotic events being 3.7% [3].

The arterial thrombosis incidents are relatively rare but significant. The rates of ischemic stroke and acute coronary syndrome (ACS)/myocardial infarction (MI) found in COVID-19 affected ICU patients in Italy were 2.5% and 1.1%, respectively. Overt disseminated intravascular coagulation (DIC) was present in 2.2% patients [4]. According to the current data, the combined arterial and venous thrombotic events in the COVID-19 affected ICU patients are up to 30% despite being on pharmacological thromboprophylaxis, with thrombotic events resulting in an increased risk of mortality around 5.4 times [5]. SARS-COV2 infects the monocytes, macrophages, and vascular endothelial cells. Infected vascular endothelial cells result in cellular damage and induce apoptosis, resulting in decreased antithrombotic activity on the vessels’ luminal surface. Healthy endothelial cells release nitric oxide, which prevents leukocyte and platelet adhesion, and inflammatory cell migration into the vessel wall, thus suppressing inflammation and apoptosis. Therefore, the damaged endothelium cannot perform these actions, resulting in a procoagulant state.

According to the series of autopsy findings, the incidence of thrombus formation in the pulmonary vasculature is approximately nine times higher in COVID-19 infected patients compared to those with influenza [6]. Lax et al. performed autopsies in 11 patients and reported thrombus formation in small and medium-sized pulmonary arteries despite the absence of clinical presentation of thromboembolism [7]. The infected monocytes and macrophages, on the other hand, express the tissue factor on their surfaces, initiating a coagulation cascade [8]. Therefore, in addition to the deep venous thrombosis resulting in an embolic event, in situ thrombus formation in the arteries can be a significant factor for pulmonary dysfunction. Despite anticoagulation, a high number of patients with ARDS secondary to COVID-19 developed life-threatening thrombotic complications, mostly pulmonary embolism [9]. We present a case of COVID positive patient developing MI in a hospital setting despite being on multiple anticoagulants to support this notion.

## Case presentation

A 51-year-old, Middle-Eastern male diabetic patient presented to the emergency room with complaints of sudden onset left leg pain, paresthesia, and swelling for one day. The patient denied any fever, chills, body aches, cough, or chest pain. No personal history of surgery, allergies, or malignancy was reported. The patient was a nonsmoker, denied the use of alcohol and illicit drugs. He lived with his wife and children, none of whom have exhibited COVID symptoms. His vital signs included a blood pressure of 134/86 mmHg, pulse rate of 117 beats/min, respiratory rate of 20/min, oximetry 97% on room air, and a temperature of 36.5°C (98.1°F). Physical examination was normal in the right leg, but the left leg was cool to touch from forefoot to mid-calf, with noticeable mottling over the forefoot and a nonpalpable dorsalis pedis. The patient was started on therapeutic enoxaparin and diltiazem in the ED. An electrocardiogram (EKG) completed in the emergency room was consistent with atrial fibrillation.

**Figure 1:**
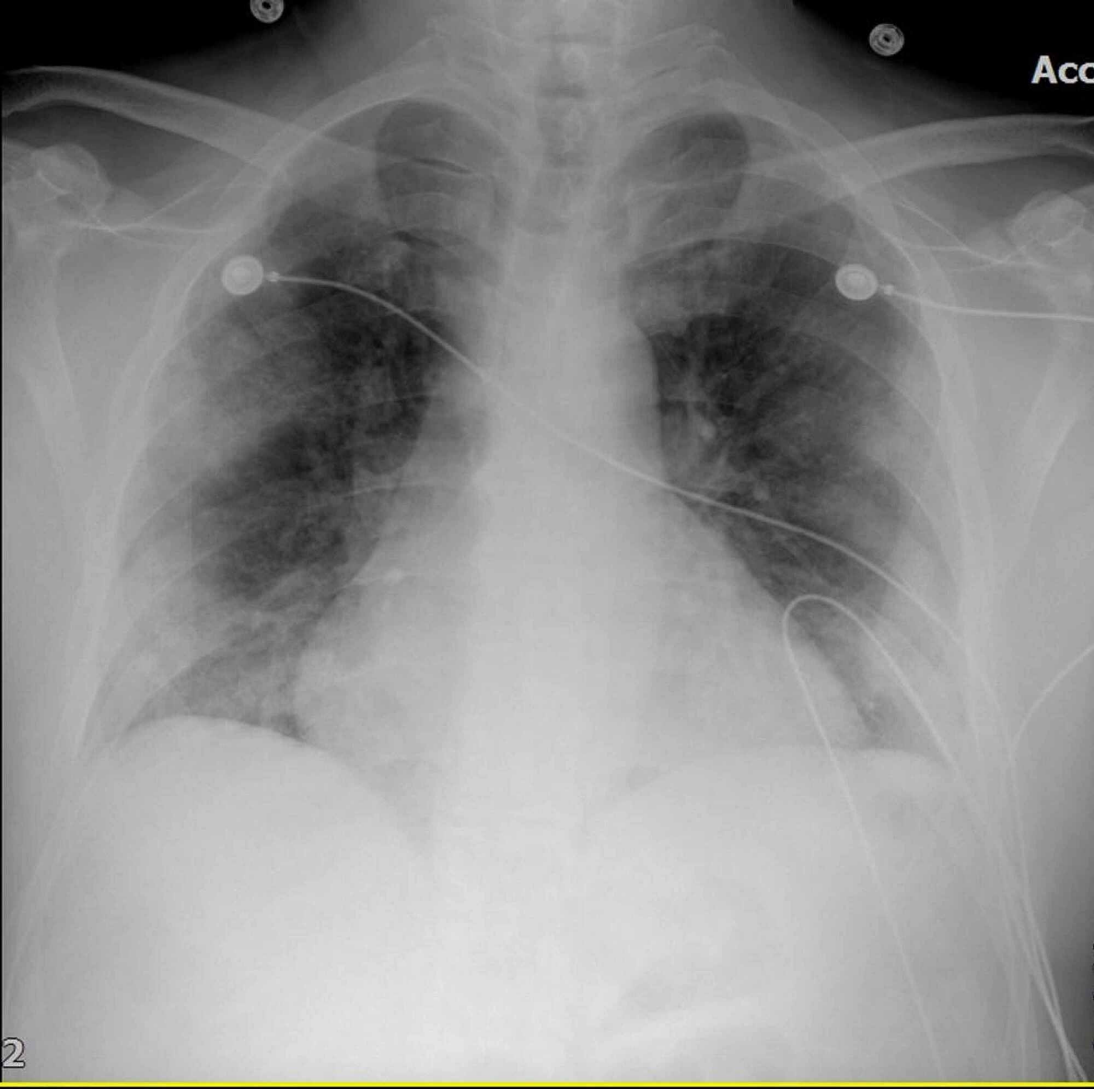
Chest X-ray showing bilateral ground glass infiltrate and COVID pneumonitis.

**Figure 2:**
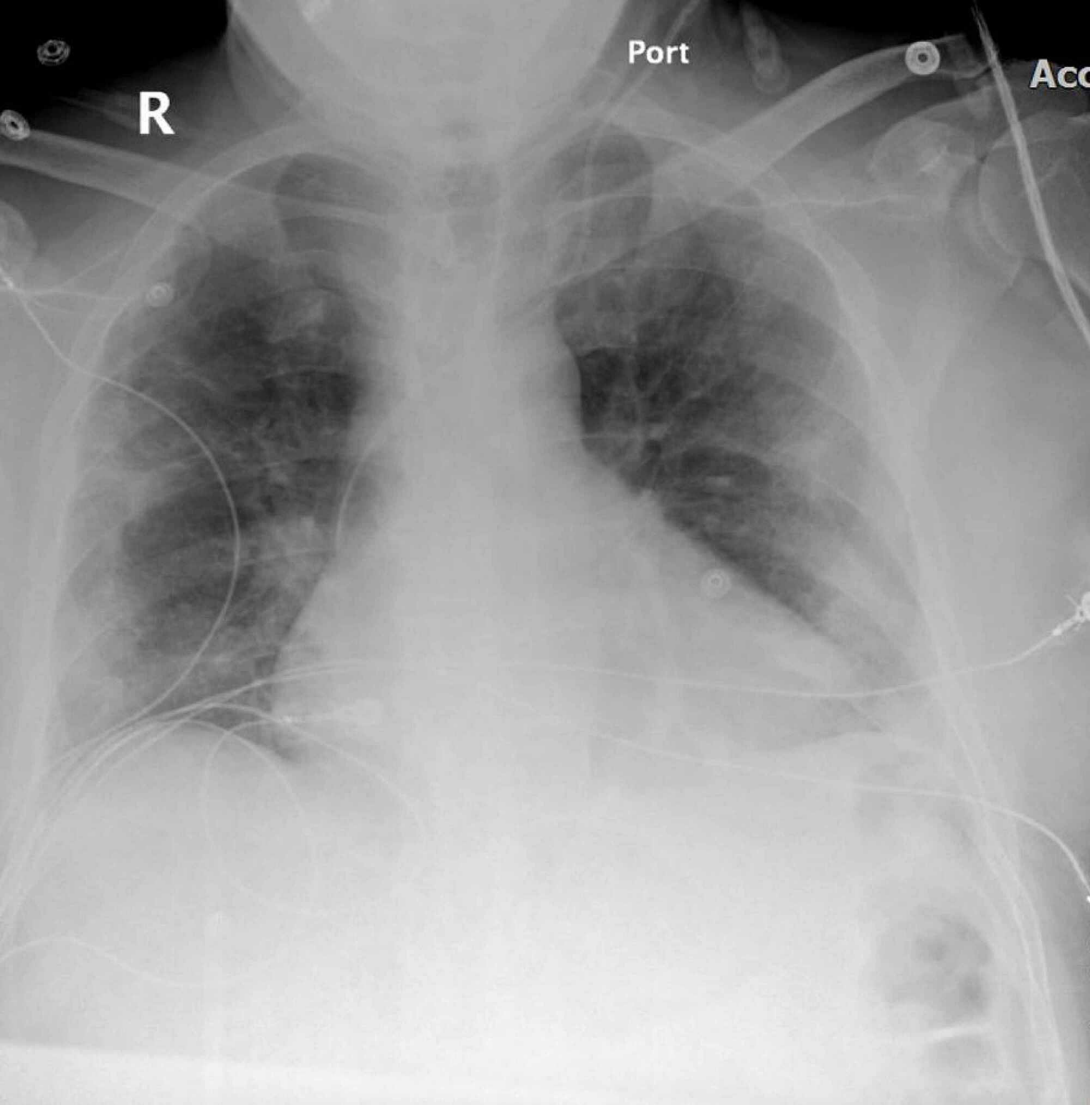
Chest X-ray showing cardiomegaly.

**Figure 3:**
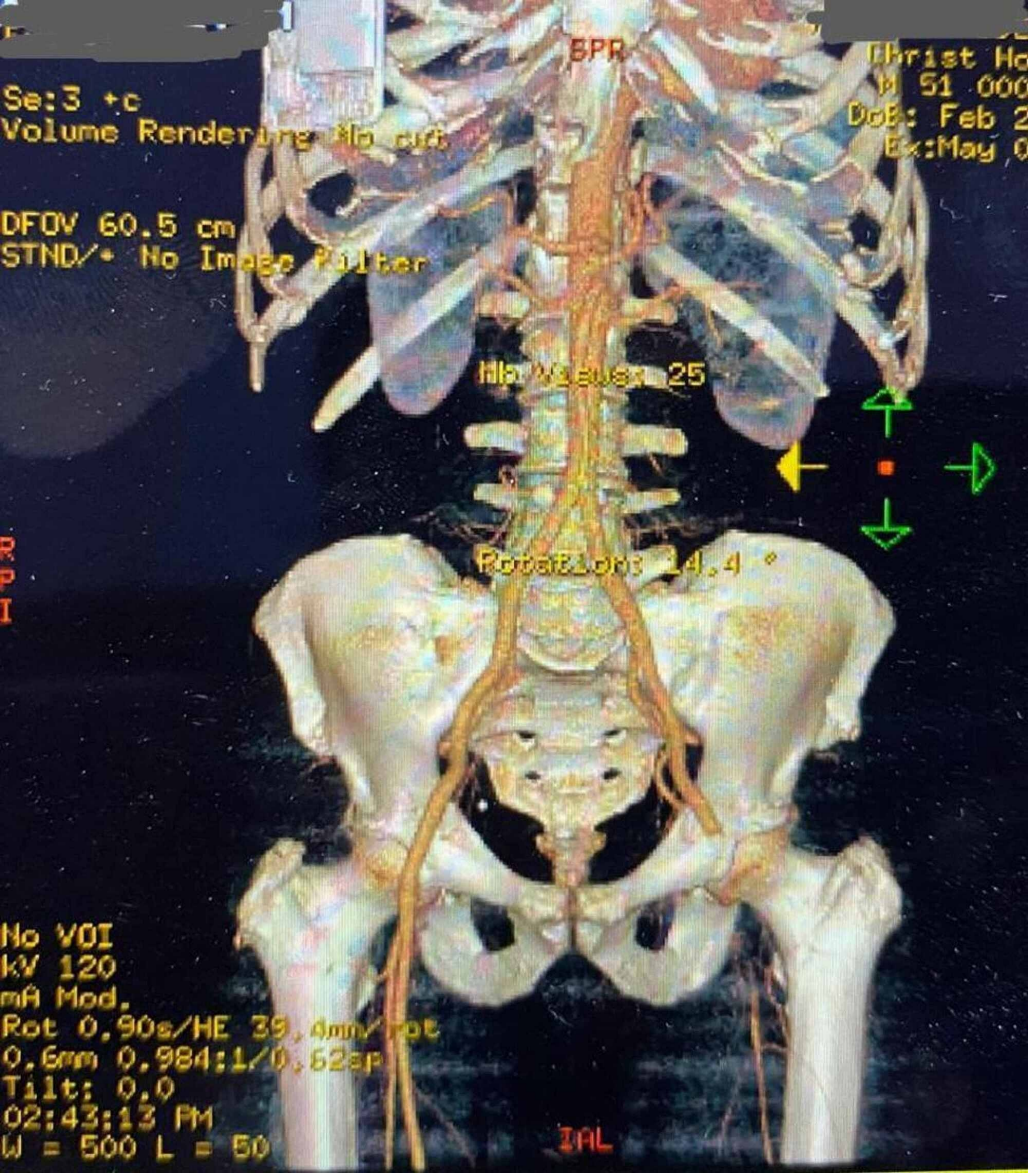
CT angiography showing an abrupt cutoff at the left common femoral artery.

**Figure 4:**
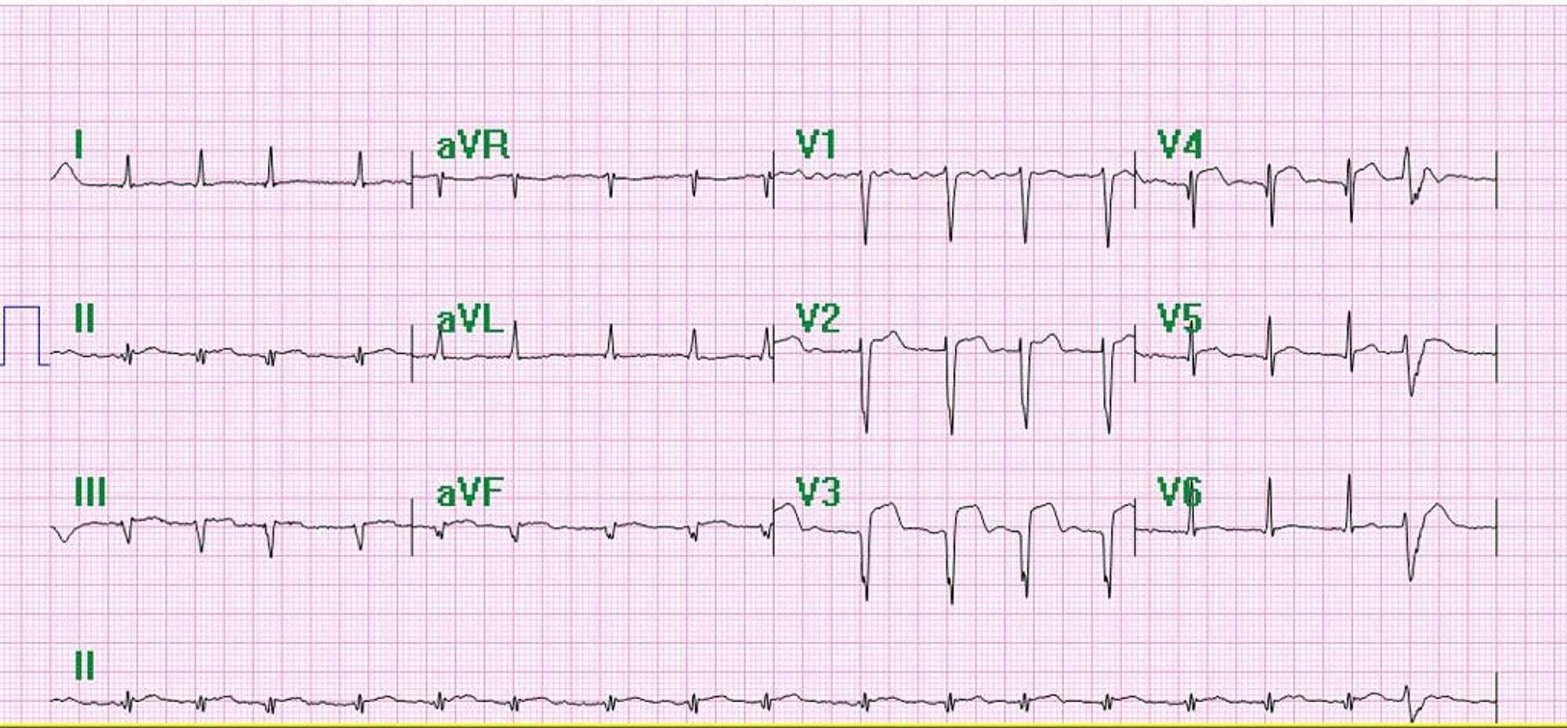
EKG showing absence of a-waves, irregular R-R intervals and consistent with atrial fibrillation. EKG, electrocardiogram

Chest X-ray showed bilateral pulmonary infiltrates beginning peripherally and COVID pneumonitis (Figures 1-2). Considering the patient’s status, we had to follow strict triage and committee approval to perform any procedures. Abnormal laboratory findings included elevated erythrocyte sedimentation rate (ESR)/C-reactive protein (CRP), blood sugar of 260 mg/dL, D-dimer at 3600, and brain natriuretic peptide (BNP) levels of 2750. Troponin was 0.0250 ng/mL. A CT of the left lower extremity was performed, which showed an abrupt cutoff at the left common femoral artery, indicating a poor prognosis (Figure 3). Clot removal was done in the catheter lab. An ekosonic endovascular system (EKOS) catheter with repeat thrombectomy plans was placed, aiding in deeper penetration of thrombolytic agents and decreasing the dose of tissue plasminogen activator (tPA) required and the likelihood of bleeding. Intravenous heparin infusion was continued overnight via tPA catheter with EKOS to see if flow in the left lower extremity can be re-established. On examination, no pulses were palpable, and only femoral flow could be appreciated on Doppler Ultrasonography. The patient underwent a mechanical thrombectomy and was loaded with aspirin/clopidogrel, heparin drip, and enoxaparin. Despite being on triple anticoagulation, the patient had new-onset ST elevations in V3 on 12 lead EKG, and troponin level was elevated at 12.4 (Figure 4). On angiography, the patient was found to have MI with occluded mid-left anterior descending, most likely from acute on chronic thrombosis related to the patient’s COVID-19 status. Despite ballooning, the flow could not be re-established. The patient was clinically stable, so the intervention had to be stopped as there was a high risk of bleeding because of tPA. He was kept on long-term protective anticoagulation-triple therapy (an oral anticoagulant and dual antiplatelet therapy) and received pulmonary care for COVID infection. The patient was discharged on long-term triple anticoagulation and COVID precautions with scheduled retesting and follow-up.

## Discussion

Coronavirus is being considered the respiratory virus but other presentations are also possible, and some are frequently emerging [10-11]. Here we present a case to support this notion where the patient presented with no respiratory complaint; instead, the COVID positive status leads to the thromboembolic phenomenon.

Our patient was a 51-year-old male who presented with tachycardia and numbness in the left lower extremity. The patient had no past history of hypercoagulable disorder. During the hospital stay, the patient had acute MI despite being on triple anticoagulation, which was presumed to be due to the patient’s positive COVID-19 status. The overall association of COVID with thrombotic events is not well defined, but it is associated with endothelial dysfunction, inflammation, activation of cytokines and inflammatory mediators, and stasis [12]. Data from such patients’ laboratory results showed thrombocytopenia, elevated D-dimer levels, prolonged prothrombin time, and overall activation of the coagulation cascade, but the exact mechanism was unknown [1]. Not only does COVID lead to an imbalance in the pro and antithrombotic states of the body, but it creates an overall picture of disseminated intravascular coagulation and predisposes to both arterial and venous thromboembolism [3].

In a single-center cohort study, Middeldorp et al. reported objectively confirmed venous thromboembolism (VTE) incidence rates of 16%, 33%, and 42% at days 7, 14, and 21, respectively, among hospitalized patients with COVID-19, despite receiving thrombosis prophylaxis [13]. Another study reports the incidence of thrombotic events in COVID patients to be as high as 31%. However, sufficient data are not available to support whether prophylactic anticoagulation in COVID-positive hospitalized patients will improve their clinical outcomes or not, and more research will be required to prove that [14].

Cardiac involvement can be due to viral injury of cardiac myocytes. COVID-19 causes systemic inflammation and increased coronary blood flow which can lead to rupture of plaque due to increased shear stress [15]. Our patient fortunately did well, and he was successfully discharged on long-term anticoagulation. Physicians must be aware of the fact that cardiovascular complications must be considered in the COVID-19 patients, even with minimal risk factors for heart disease.

## Conclusions

SARS-CoV-2 infection can lead to a procoagulant state leading to arterial and venous thrombosis. Patients with COVID-19 and MI may require a long period of hospital stay, demanding multiple anticoagulants to overcome critical clinical conditions.
